# Absence of long-term changes in urine biomarkers after AKI: findings from the CRIC study

**DOI:** 10.1186/s12882-022-02937-x

**Published:** 2022-09-13

**Authors:** Ian E. McCoy, Jesse Y. Hsu, Joseph V. Bonventre, Chirag R. Parikh, Alan S. Go, Kathleen D. Liu, Ana C. Ricardo, Anand Srivastava, Debbie L. Cohen, Jiang He, Jing Chen, Panduranga S. Rao, Anthony N. Muiru, Chi-yuan Hsu

**Affiliations:** 1 Division of Nephrology, University of California San Francisco, Box 0532, 500 Parnassus Ave., MUW418, 94143-0532 San Francisco, CA USA; 2 Division of Biostatistics, University of Pennsylvania, Philadelphia, PA USA; 3 Division of Renal Medicine, Brigham and Women’s Hospital and Harvard Medical School, Boston, MA USA; 4 Division of Nephrology, Johns Hopkins University, Baltimore, MD USA; 5 Division of Research, Kaiser Permanente Northern California, Oakland, CA USA; 6 Division of Nephrology, University of Illinois, Chicago, IL USA; 7 Division of Nephrology and Hypertension, Center for Translational Metabolism and Health, Institute for Public Health and Medicine, Northwestern University Feinberg School of Medicine, Chicago, IL USA; 8 Division of Nephrology, University of Pennsylvania, Philadelphia, PA USA; 9 Department of Epidemiology, Tulane University, New Orleans, Louisiana, USA; 10 Division of Nephrology, Tulane University, New Orleans, Louisiana, USA; 11 Division of Nephrology, University of Michigan, Ann Arbor, MI USA

**Keywords:** Acute kidney injury, Chronic kidney disease, Biomarkers

## Abstract

**Background:**

Mechanisms by which AKI leads to CKD progression remain unclear. Several urine biomarkers have been identified as independent predictors of progressive CKD. It is unknown whether AKI may result in long-term changes in these urine biomarkers, which may mediate the effect of AKI on CKD progression.

**Methods:**

We selected 198 episodes of hospitalized AKI (defined as peak/nadir inpatient serum creatinine values ≥ 1.5) among adult participants in the Chronic Renal Insufficiency Cohort (CRIC) Study. We matched the best non-AKI hospitalization (unique patients) for each AKI hospitalization using pre-hospitalization characteristics including eGFR and urine protein/creatinine ratio. Biomarkers were measured in banked urine samples collected at annual CRIC study visits.

**Results:**

Urine biomarker measurements occurred a median of 7 months before and 5 months after hospitalization. There were no significant differences in the change in urine biomarker-to-creatinine ratio between the AKI and non-AKI groups: KIM-1/Cr + 9% vs + 7%, MCP-1/Cr + 4% vs + 1%, YKL-40/Cr + 7% vs -20%, EGF/Cr -11% vs -8%, UMOD/Cr -2% vs -7% and albumin/Cr + 17% vs + 13% (all *p* > 0.05).

**Conclusion:**

In this cohort of adults with CKD, AKI did not associate with long-term changes in urine biomarkers.

**Supplementary Information:**

The online version contains supplementary material available at 10.1186/s12882-022-02937-x.

## Introduction

Acute kidney injury (AKI) complicates 10–20% of hospitalizations [1–3] and is associated with the development and progression of chronic kidney disease (CKD) [3–10]. The mechanisms by which AKI results in CKD still remain unclear [11]. AKI can cause acute loss of nephron mass and may also lead to faster CKD progression [12, 13]. We hypothesized that AKI may increase biomarkers predictive of CKD progression, which may provide pathophysiological insight into how AKI accelerates CKD progression.

Urine biomarkers of kidney tubular injury (KIM-1), inflammation (monocyte chemoattractant protein 1; MCP-1 and human cartilage glycoprotein-40; YKL-40), tubular health (epidermal growth factor; EGF and uromodulin; UMOD), and glomerular and tubular disease (albumin) have each been associated with CKD progression [14–19] and are known to rise, at least transiently, after AKI [20–24]. If AKI has a differential long-term effect on these biomarkers (e.g., markers of inflammation are chronically increased while markers of tubular health are unaffected), such results may provide additional mechanistic insights into the long-term effects of AKI on CKD progression.

We recently showed that plasma levels of KIM-1 and Tumor Necrosis Factor Receptors 1 and 2 (TNFR1 and TNFR2) show long-term increases after AKI [25]. Leveraging the same study population and design, here we investigate whether and how among patients with CKD, an episode of AKI is associated with long-term changes in several urine biomarkers (KIM-1, MCP-1, YKL-40, EGF, UMOD, and albumin), which reflect additional pathophysiological pathways.

## Methods

The study design used to assemble this cohort has been previously described [25]. In brief, we studied participants in the Chronic Renal Insufficiency Cohort (CRIC) Study, an ongoing multicenter prospective observational cohort study of adults with CKD [26].﻿ CRIC study participants attended annual in person visits where samples of blood and urine were taken and had mid-year telephone contacts to update medical history. The CRIC Study protocol was approved by the institutional review boards of all participating centers and is in accordance with the Declaration of Helsinki. All participants provided written informed consent. Only CRIC study participants who were alive and active in the study after July 2013 were selected for the current study population since it was only after this date that inpatient serum creatinine readings were systematically captured to define presence or absence of AKI. We did not include hospitalizations after December 2019.

Adapting from the KDIGO definition [27, 28], we defined AKI hospitalizations as those with peak/nadir inpatient serum creatinine values $\ge$ 1.5. To be classified as a non-AKI hospitalization, all three of the following criteria had to have been met: peak/nadir inpatient serum creatinine < 1.2 *and* peak minus nadir inpatient serum creatinine < 0.3 mg/dL *and* peak inpatient/most recent outpatient study visit serum creatinine < 1.5. Hospitalizations that did not meet criteria for AKI or non-AKI were excluded in an effort to achieve greater separation. We also excluded hospitalizations after which end-stage kidney disease (ESKD) developed prior to the next scheduled annual post-discharge CRIC study visit. Hospitalizations were only eligible if there were CRIC study visits with both plasma and urine sample collection within two years prior to admission and within one year after discharge [25].

199 AKI hospitalizations and 1534 non-AKI hospitalizations met the inclusion/exclusion criteria. We matched each AKI hospitalization to a non-AKI hospitalization (patients could only contribute one hospitalization to the matching) as previously described [25] using pre-hospitalization eGFR, urine protein-to-creatinine ratio (UPCR), days between hospital discharge and next CRIC visit, diabetes status, age, sex, and days between hospital admission and prior CRIC visit. Missing pre-hospitalization eGFR precluded matching one AKI patient. 198 matches (198 AKI patients and 198 non-AKI patients) comprised our final cohort.

Spot urine samples collected at study visits were placed on ice immediately after collection. Within one hour of collection, they were centrifuged for five minutes at 2000 g in a refrigerated centrifuge set at 4 °C. Supernatants were then frozen locally at either -20 or -80 °C before being shipped to the central lab on dry ice, where they were stored at -80 °C until they were thawed for measurement. Urine biomarkers (KIM-1, MCP-1, YKL-40, EGF, and UMOD) were measured using a multiplex U-PLEX assay on the Meso Scale Discovery platform (Meso Scale Discovery, Gaithersburg, MD), albumin was measured by an immunoturbidimetric method, and urine creatinine was measured by the Jaffe colorimetric method (Randox, Crumlin UK) at Johns Hopkins Hospital. These biomarkers were chosen based on prior research showing that these biomarker levels change acutely in the setting of AKI [20, 24] and are also associated with CKD [14, 19]. 32 samples for YKL-40 resulted as above the upper detection limit of the assay (5 × 10^5^ pg/mL), so 5 × 10^5^ pg/mL was imputed as the result for these samples.

We presented descriptive statistics as proportions, means and standard deviations, or medians and interquartile ranges. We used paired t tests and Wilcoxon signed-rank tests (for means and medians, respectively) for continuous variables and McNemar’s tests for categorical variables to generate P values presented in Tables 1 and 2. All biomarker-to-creatinine distributions were right-skewed so values were log-transformed for analysis. For the primary analysis accounting for correlations among matched pairs of patients comparing changes in biomarker-to-creatinine ratios between AKI and non-AKI groups (Table 3), we used a linear mixed effects model including the fixed effects of AKI, change between the pre/post-hospitalization visits, and their interaction (AKI with change between visits), and random effects of match ID and participant ID: Y = Natural log of biomarker-to-creatinine ratio = ß0 + ß1[AKI] + ß2[Post-hospitalization] + ß3[AKI*Post-hospitalization] + random intercepts for participant ID and matched pair, where ß0 is the mean of the log of the non-AKI pre-hospitalization measurement, and AKI and Post-hospitalization are binary variables indicating whether the measurement was measured in a patient with AKI and at the post-hospitalization visit. This model estimates both the percent change in urine biomarker concentrations between pre- and post-hospitalization measurements (percent change for the non-AKI group given by 100*(e^ß2 – 1) and percent change for the AKI group given by 100*(e^ß2 * e^ß3 – 1)) and the ratio (AKI vs non-AKI) of those pre/post-hospitalization percent changes (given by 100*(e^ß3 – 1)). Since patients had already been matched on important confounders during cohort assembly, no statistical adjustment for confounders was performed.

To evaluate the effect of AKI on long-term eGFR loss after the post-hospitalization visit, we used a linear mixed effects model with eGFR as the outcome and with time as a continuous variable (Y = eGFR = ß0 + ß1[AKI] + ß2[years after post-hospitalization visit] + ß3[AKI*years] + random intercepts for participant ID and matched pair). This analysis only included eGFR values measured after hospitalization.

All analyses were performed using R 4.0.2 (R Core Team (2020) R: A language and environment for statistical computing. R Foundation for Statistical Computing, Vienna, Austria. https://www.R-project.org/).

## Results

### Pre-hospitalization and hospitalization characteristics

The 198 patients with AKI during hospitalization and 198 patients without AKI during hospitalization were well-matched on all pre-hospitalization characteristics, including eGFR (mean 48 vs 48 mL/min/1.73m^2^) and UPCR (median 0.24 vs 0.26 g/g) (Table 1), ascertained at the most recent annual CRIC study visit before hospitalization.

**Table 1:** Pre-hospitalization, hospitalization, and post-hospitalization characteristics

*Pre-Hospitalization* *Characteristics*	AKI Patients (*n* = 198)	Non-AKI Patients (*n* = 198)	*P*-Value
**Age, yrs**	67 [60–73]	67 [61–73]	0.48
**Male**	61%	64%	0.21
**Race/Ethnicity**			
Hispanic	10%	8%	0.67
Non-Hispanic Black	47%	49%	
Non-Hispanic White	40%	40%	
**Diabetes mellitus**	68%	68%	1.00
**Estimated GFR, mL/min/1.73m**^**2**^	47 [35–57]	46 [34–57]	0.10
**Urine protein to creatinine ratio, g/g**	0.24 [0.10–0.99]	0.26 [0.08–0.90]	0.48
**Systolic blood pressure, mmHg**	126 [113–142]	127 [114–139]	0.50
**Days between pre-hospitalization measurement and admission**	221 [138–291]	220 [123–283]	0.06
***Hospitalization Characteristics***			
**AKI Stage**^a^			
Stage 1	66%	N/A	N/A
Stage 2	28%	N/A	N/A
Stage 3	7%	N/A	N/A
**Cardiovascular Hospitalization**	25%	27%	0.73
**Infection-related Hospitalization**	14%	8%	0.12
***Post-Hospitalization Annual CRIC Visit Characteristics***			
** Estimated GFR, mL/min/1.73m**^**2**^	42 [31–55]	46 [32–59]	< 0.01
** Urine protein to creatinine ratio, g/g**	0.31 [0.11–1.06]	0.27 [0.10–0.91]	0.55
** Systolic blood pressure, mmHg**	128 [113–143]	126 [115–138]	0.47
** Days between discharge and post-hospitalization measurement**	161 [93–229]	160 [93–247]	0.20
** Days between pre-hospitalization and post-hospitalization measurements**	369 [348–413]	365 [349–390]	< 0.01

Median [IQR] for continuous variables or percentages for categorical variables. Missingness for pre-hospitalization variables was 9 patients for UPCR, 2 patients for SBP, and 1 patient for ACEi/ARB use. Missingness for post-hospitalization variables was 5 patients for eGFR, 23 patients for UPCR, 4 patients for SBP, and 3 patients for ACEi/ARB use.

^a^Percentages do not sum to 100% due to rounding

Pre-hospitalization biomarker measurement occurred about 7 months before hospital admission in both groups (median 221 days [IQR 138–291] in the AKI group versus 220 days [IQR 123–283] in the non-AKI group; *p* = 0.06). Post-hospitalization biomarkers were measured about 5 months after hospital discharge (median 161 days [IQR 93–229] in the AKI group versus 160 days [IQR 93–247] in the non-AKI group; *p* = 0.20; Table 1). Roughly one quarter of hospitalizations were due to cardiovascular causes in both the AKI and non-AKI groups [25]. Two thirds of AKI cases were mild (stage 1), and none required dialysis.

Urine biomarker concentrations normalized to urine creatinine concentrations were similar before hospitalization in the AKI and non-AKI groups (Table 2), though the AKI group had borderline statistically significantly higher median pre-hospitalization MCP-1/Cr (223 pg/mg versus 196 pg/mg in the non-AKI group, *p* = 0.04 without adjustment for multiple hypothesis testing). These results were similar when the biomarker concentrations were not normalized to urine creatinine (Table S1).

**Table 2:** Urine biomarker-to-creatinine ratios

Result	AKI Hospitalization (*n* = 198)	Non-AKI Hospitalization (*n* = 198)	*P*-Value
KIM-1/Cr (pg/mg)			
Pre-hospitalization	771 [465–1132]	646 [402–1043]	0.11
Post-hospitalization	744 [466–1280]	709 [403–1203]	0.08
Raw change (and % change)	40 [-210–328] (9%)	35 [-112–229] (7%)	0.88
MCP-1/Cr (pg/mg)			
Pre-hospitalization	223 [136–361]	196 [128–288]	0.04
Post-hospitalization	220 [136–386]	184 [125–309]	< 0.01
Raw change (and % change)	9 [-107–140] (4%)	1 [-60–65] (1%)	0.46
YKL-40/Cr (pg/mg)			
Pre-hospitalization	596 [232–2308]	534 [211–2351]	0.99
Post-hospitalization	709 [179–2830]	520 [201–1703]	0.20
Raw change (and % change)	16 [-353–1526] (7%)	-46 [-712–388] (-20%)	0.06
EGF/Cr (pg/mg)			
Pre-hospitalization	1975 [1166–2947]	1818 [1240–3010]	0.78
Post-hospitalization	1768 [1027–2838]	1720 [1111–2730]	0.62
Raw change (and % change)	-164 [-574–196] (-11%)	-115 [-490–180] (-8%)	0.63
UMOD/Cr (ug/mg)			
Pre-hospitalization	10.52 [5.48–18.10]	10.35 [5.01–22.78]	0.63
Post-hospitalization	9.31 [4.98–19.66]	10.20 [5.44–20.95]	0.84
Raw change (and % change)	-0.16 [-6.80–6.34] (-2%)	-0.87 [-6.90–4.72] (-7%)	0.61
Albumin/Cr (mg/g)			
Pre-hospitalization	145 [28–740]	141 [29–646]	0.26
Post-hospitalization	163 [35–765]	128 [35–712]	0.34
Raw change (and % change)	7 [-63–123] (17%)	5 [-74–100] (13%)	0.99

Values given in median [IQR]. *P* values for changes correspond to raw changes, not percent changes

### Urine biomarker changes from pre-hospitalization to post-hospitalization

There were no statistically significant changes in the levels of most biomarkers over time from pre- to post-hospitalization, neither in the AKI group nor in the non-AKI group. The only change in urine biomarker-to-creatinine ratio from pre-hospitalization to post-hospitalization that reached statistical significance at the *p* < 0.05 threshold was EGF/Cr (decreased by about 10% in both groups, *p* < 0.001 for difference from pre- to post-hospitalization).

### Impact of AKI

AKI was not associated with significant differences in the change in urine biomarker-to-creatinine ratio from the pre- to the post-hospitalization measurements: KIM-1/Cr + 9% (AKI group) vs + 7% (non-AKI group), MCP-1/Cr + 4% vs + 1%, YKL-40/Cr + 7% vs -20%, EGF/Cr -11% vs -8%, UMOD/Cr -2% vs -7% and albumin/Cr + 17% vs + 13% (all *p* > 0.05, Table 2). Results were similar for the raw biomarker concentrations not normalized to urine creatinine concentration (Table S1). In the mixed effects model accounting for matched pairs, no differences in change in urine biomarker-to-creatinine ratios were statistically significant (all *p* > 0.05, Table 3). When AKI was evaluated by stage, there were still no significant changes for any urine biomarker with AKI stage 1, stage 2, or stage 3 (Table S2).

**Table 3:** Urine biomarker-to-creatinine ratio changes in mixed effects models

Result	Ratio of percent change in biomarker concentration in AKI vs non-AKI [95% Confidence Interval]	*P*-Value
KIM-1/Cr	1.015 [0.905–1.139]	0.79
MCP-1/Cr	1.049 [0.905–1.217]	0.52
YKL-40/Cr	1.429 [0.781–2.614]	0.25
EGF/Cr	0.965 [0.896–1.039]	0.34
UMOD/Cr	1.008 [0.832–1.215]	0.95
Albumin/Cr	1.044 [0.850–1.281]	0.68

### Post-hospitalization estimated glomerular filtration rate

**Fig. 1:**
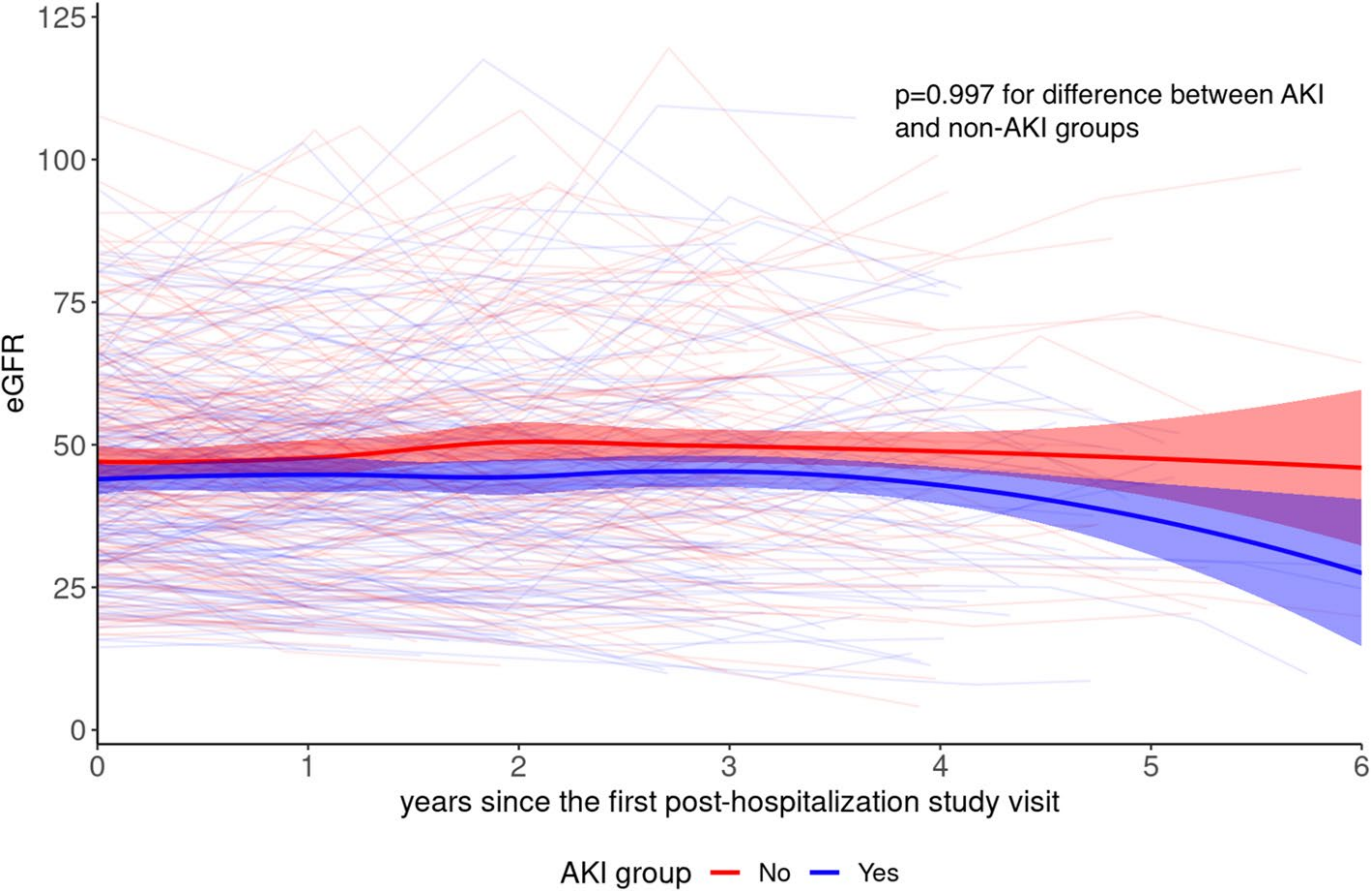
Post-hospitalization changes in eGFR. Legend: Each faded line shows the eGFR measurements for an individual patient. The bright lines show non-parametric smooth eGFR trajectories for each group. *P* value from linear mixed effects model

Given these results, we examined eGFR trajectory after hospitalization. 393 patients (of the 396 in our cohort) had a total of 1293 eGFR measurements after hospitalization (median 3 measurements per patient, IQR 2–4). At the post-hospitalization visit, mean eGFR had dropped significantly further in the AKI group (from 48 to 44 ml/min/1.73m^2^ in the AKI group compared to from 48 to 47 ml/min/1.73m^2^ in the non-AKI group; difference in differences -3 ml/min/1.73m^2^, *p* < 0.01). However, over mean follow-up of 4.0 (SD 1.5) years after the post-hospitalization visit, there was no difference in eGFR trajectory between the AKI and non-AKI groups (Fig. 1; *p* = 0.997).

## Discussion

We found that, among patients with pre-existing CKD, AKI was not associated with long-term changes in urine KIM-1, MCP-1, YKL-40, EGF, UMOD, and albumin. Overall, urinary biomarkers were stable over the approximately 1-year period between measurements despite all participants experiencing intervening hospitalization (only EGF/Cr showed a significant decrease with time, though this decrease did not differ between those with and without AKI).

These results contrast with our work measuring plasma biomarkers in this same cohort at the same time [25], which showed that AKI was associated with elevations in plasma KIM-1, TNFR1, and TNFR2. There may be multiple possible explanations for these contrasting results.

One, the effect by which AKI increases CKD progression may not be mediated by any of the pathophysiological pathways captured by the urine biomarkers we selected to examine. In contrast, the plasma biomarkers such as TNFR1 and TNFR2 may capture a more relevant pathophysiological mechanism.

Two, the same biomarker measured in the urine may be less informative than when it is measured in the plasma. This appears to be the case for KIM-1 as we noted in CRIC that AKI was associated with a subsequent increase in plasma levels [25], but without any change in urine levels of KIM-1. It has been suggested that plasma KIM-1 reflects time-averaged tubular injury, whereas urine KIM-1 may be more variable due to fluctuations in urinary excretion over time [29]. Data from the ACCORD trial showed that urine KIM-1 was not associated with CKD progression [30], but plasma KIM-1 was strongly associated [31]. Although some investigators have suggested broadly that plasma biomarkers are superior to urine biomarkers for CKD progression [32], this may vary from biomarker to biomarker. For instance, MCP-1 in the urine has been repeatedly associated with CKD progression [14–16], while MCP-1 in the plasma has not [33, 34]. Others like EGF are virtually undetectable in plasma, but well associated with CKD progression in the urine.

Three, the study may be underpowered, and the mostly mild AKI seen in this cohort may have only a modest effect, which may be difficult to detect from a study of this size. Prior studies that demonstrated a significant effect of mild to moderate AKI on CKD progression may have overestimated the AKI effect due to inadequate adjustment for significant confounding from pre-AKI proteinuria and eGFR slope [35, 36]. Our analysis of CKD progression here also did not show any effect of AKI on long-term eGFR trajectory after the initial drop in eGFR (Fig. 1). Thus, the effects of AKI of this severity may be truly mild. Arguing against this possibility is the fact that we previously detected significant increases with AKI in plasma KIM-1, TNFR1, and TNFR2 in this same cohort, measured at the same time as the urine biomarkers in the present study [25]. More research is needed to define the characteristics and severity of AKI episodes that are likely to affect CKD progression.

A fourth possibility may be that the AKI-associated increases in plasma biomarkers we previously found in this cohort [25] were confounded by decreases in eGFR following AKI. In other words, plasma biomarker levels may be increased post-AKI due to reduced clearance post-AKI rather than increased production. We think this possibility is unlikely given the size of the measured plasma biomarkers (90 kDa for KIM-1 [29], 55 kDa for TNFR1 [37], and 80 kDa for TNFR2 [38]), but we cannot rule out the possibility that smaller biomarker fragments could have been detected by our assays. In addition, these biomarkers have been consistently associated with future CKD progression – independent of baseline eGFR [31, 39–41], which suggests that biomarker concentrations are not determined solely by glomerular clearance.

Our study adds important information to the literature on long-term changes in urine biomarker concentrations measured months before and months after an episode of AKI. Much of the prior literature associating urine biomarkers with AKI only have biomarker measurements at a single timepoint, often lacking any pre-AKI biomarker measurements [23, 42, 43], and those that do have biomarker measurements at multiple timepoints are often measured hours to days before and after AKI [21, 44]. We know of only two prior studies assessing long-term changes in novel urine biomarkers after AKI [20, 22].

In the FRAIL-AKI study, Cooper et al. associated AKI with significant long-term (seven years) increases in urine KIM-1, IL-18, NGAL, and L-FABP, despite no differences in eGFR or albuminuria in a pediatric cardiac surgery population (N = 30 with AKI and 18 without AKI) [20]. Their AKI episodes were more severe (2/3 of their cohort had stage 2 or 3 AKI), but our analysis of AKI by stage did not find an association with long-term urine biomarker changes at any stage of AKI (Table S2). Perhaps the most likely explanation for the discrepant results is the difference in the patient population studied (children without CKD in the FRAIL-AKI study versus adults with CKD in our study). It is conceivable that the effects of AKI are easier to detect in a population without CKD than in a population in which background CKD has already caused elevations in urine KIM-1 (lower signal to noise ratio).

A second study, which evaluated a similar population as CRIC (adults with CKD in the SPRINT trial) had results which were more concordant with ours. Bullen et al. found no association between AKI (defined by discharge summaries) and long-term (four years) changes in urine KIM-1, UMOD, MCP-1, or beta-2 microglobulin. They did find that AKI was associated with mildly significant greater percent increases in urinary YKL-40 (*p* = 0.03), NGAL (*p* = 0.02), alpha-1 microglobulin (*p* = 0.009), and IL-18 (*p* = 0.03), but no adjustments were performed for baseline differences between those with and without AKI [22].

Our study has several strengths. The structure of CRIC with regular urine sample collection at annual study visits allowed us to ascertain pre-AKI biomarker levels, which are often not available in AKI studies. This structure also allowed us to repeat biomarker measurements several months after hospital discharge, while such long-term follow-up is unavailable in many AKI studies. The collection of detailed serum creatinine information from intervening hospitalizations in CRIC allowed us to minimize misclassification, which can be problematic in studies that rely on administrative billing codes to ascertain AKI [45]. Our strict definitions for both AKI and non-AKI based on laboratory information exclude borderline patients who were not clearly AKI or non-AKI and thus further minimize misclassification. This study is the first report (to our knowledge) evaluating the association of AKI with long-term changes in urine EGF. Finally, our data included all-cause AKI, while many other AKI studies are restricted to a particular type of AKI (e.g., post-cardiac surgery AKI) [20, 44] since surgery is one of the few causes of AKI that is predictable and thus allows sample collection both before and after AKI.

Limitations of our study should also be noted. As discussed above, we may have been underpowered to detect small effects of AKI on changes in these urine biomarkers, but none of the biomarkers showed AKI-associated changes of even borderline significance (Table 3). Since most of the AKI in our cohort was mild, we may have missed the effects of more severe AKI, although our analysis by AKI stage (Table S2) did not suggest this possibility to be likely. Our creatinine-based AKI definition may be non-specific for intrinsic kidney damage versus other causes of creatinine rise such as volume depletion [46], which may have a distinct effect on these urine biomarkers [47]. All CRIC study enrollees were adults, and all had baseline CKD at study entry and only included those who volunteered for research studies. We did not have biomarker measurements during the index hospitalization coinciding with the time of occurrence of AKI. Finally, our panel of urine biomarkers is not exhaustive; other potential urine biomarkers may have picked up a signal missed by the analytes we selected.

## Conclusions

In summary, differing with our work measuring plasma biomarkers (KIM-1, TNFR1, and TNFR2) in this same cohort at the same time [25], AKI was not associated with long-term changes in urine KIM-1, MCP-1, YKL-40, EGF, UMOD, or albumin several months after hospitalization.

## Supplementary Information

**Additional file 1: Table S1.** Raw urine biomarker levels without normalization to urine creatinine. **Table S2.** Urine biomarker-to-creatinine ratio changes in mixed effects models by AKI stage.

## Data Availability

The data analyzed in the current study are available in the NIDDK Central Repository: https://repository.niddk.nih.gov/studies/cric/.
