# Supplementary material for: Absence of long-term changes in urine biomarkers after AKI: findings from the CRIC study
Source: BMC Nephrol. 2022 Sep 13;23:311. doi: 10.1186/s12882-022-02937-x (PMC9472364; doi:10.1186/s12882-022-02937-x)
Supplement: Supplementary file 1 — Additional file 1: Table S1. Raw urine biomarker levels without normalization to urine creatinine. Table S2. Urine biomarker-to-creatinine ratio changes in mixed effects models by AKI stage. [file 12882_2022_2937_MOESM1_ESM.docx]

**Supplementary Material**

**Table S1. Raw urine biomarker levels without normalization to urine creatinine**

| Result | AKI Hospitalization  (n = 198) | Non-AKI Hospitalization  (n = 198) | *P*-Value |
| --- | --- | --- | --- |
| KIM-1 (pg/mL) |  |  |  |
| Pre-hospitalization | 674 [328-1330] | 577 [294-1069] | 0.13 |
| Post-hospitalization | 641 [304-1272] | 604 [304-1160] | 0.16 |
| Raw change (and %  change) | -27 [-422-295] (-8%) | -18 [-245-327] (-2%) | 0.46 |
| MCP-1 (pg/mL) |  |  |  |
| Pre-hospitalization | 211 [116-402] | 178 [102-326] | 0.11 |
| Post-hospitalization | 204 [94-385] | 166 [91-305] | 0.03 |
| Raw change (and %  change) | -20 [-143-88] (-11%) | -12 [-93-78] (-10%) | 0.84 |
| YKL-40 (pg/mL) |  |  |  |
| Pre-hospitalization | 627 [248-1762] | 600 [143-2066] | 0.72 |
| Post-hospitalization | 602 [155-2268] | 443 [127-1552] | 0.30 |
| Raw change (and %  change) | -14 [-505-1004] (-14%) | -31 [-712-463] (-16%) | 0.08 |
| EGF (pg/mL) |  |  |  |
| Pre-hospitalization | 1565 [870-3242] | 1656 [916-3109] | 0.82 |
| Post-hospitalization | 1314 [643-2372] | 1359 [726-3057] | 0.13 |
| Raw change (and %  change) | -264 [-1130-180] (-24%) | -154 [-1077-431] (-16%) | 0.04 |
| UMOD (ng/mL) |  |  |  |
| Pre-hospitalization | 8923 [5273-15972] | 9535 [5787-16043] | 0.99 |
| Post-hospitalization | 7483 [5053-12316] | 8265 [4474-15018] | 0.21 |
| Raw change (and %  change) | -903 [-5751-2696] (-15%) | -684 [-5991-3844] (-12%) | 0.39 |
| Albumin (mg/dL) |  |  |  |
| Pre-hospitalization | 10.0 [2.6-66.5] | 11.1 [1.5-50.0] | 0.83 |
| Post-hospitalization | 11.4 [1.8-57.6] | 11.8 [2.0-59.2] | 0.59 |
| Raw change (and %  change) | 0.0 [-9.1-8.1] (0%) | 0.0 [-10.4-5.8] (0%) | 0.42 |
| Creatinine (mg/dL) |  |  |  |
| Pre-hospitalization | 98.3 [59.2-137.2] | 94.3 [59.2-142.7] | 0.74 |
| Post-hospitalization | 82.1 [54.9-120.3] | 83.8 [55.9-130.4] | 0.40 |
| Raw change (and %  change) | -6.8 [-45.6-14.2] (-9%) | -5.1 [-34.8-29.0] (-8%) | 0.18 |

Values given in median [IQR]. Median change in urine albumin was zero due to most patients having undetectable urine albumin before and after hospitalization. P values for changes correspond to raw changes, not percent changes.

**Table S2.** **Urine biomarker-to-creatinine ratio changes in mixed effects models by AKI stage**

| Result | Ratio of percent change in biomarker concentration in AKI vs non-AKI [95% Confidence Interval] | *P*-Value |
| --- | --- | --- |
| KIM-1/Cr |  |  |
| AKI Stage 1 | 1.019 [0.895-1.159] | 0.78 |
| AKI Stage 2 | 0.996 [0.836-1.185] | 0.96 |
| AKI Stage 3 | 1.068 [0.770-1.482] | 0.69 |
| MCP-1/Cr |  |  |
| AKI Stage 1 | 1.060 [0.897-1.252] | 0.50 |
| AKI Stage 2 | 1.023 [0.816-1.281] | 0.85 |
| AKI Stage 3 | 1.061 [0.695-1.620] | 0.78 |
| YKL-40/Cr |  |  |
| AKI Stage 1 | 1.548 [0.784-3.054] | 0.21 |
| AKI Stage 2 | 1.205 [0.481-3.018] | 0.69 |
| AKI Stage 3 | 1.326 [0.236-7.434] | 0.75 |
| EGF/Cr |  |  |
| AKI Stage 1 | 0.958 [0.882-1.041] | 0.31 |
| AKI Stage 2 | 1.010 [0.903-1.130] | 0.86 |
| AKI Stage 3 | 0.848 [0.687-1.047] | 0.13 |
| UMOD/Cr |  |  |
| AKI Stage 1 | 1.045 [0.845-1.293] | 0.68 |
| AKI Stage 2 | 0.995 [0.747-1.325] | 0.97 |
| AKI Stage 3 | 0.712 [0.415-1.220] | 0.22 |
| Albumin/Cr |  |  |
| AKI Stage 1 | 1.140 [0.907-1.434] | 0.26 |
| AKI Stage 2 | 0.803 [0.589-1.095] | 0.17 |
| AKI Stage 3 | 1.310 [0.732-2.345] | 0.36 |

N = 130 with AKI Stage 1, 55 with AKI Stage 2, and 13 with AKI Stage 3.
